# Macrosomia Rates in Women with Diet-Controlled Gestational Diabetes: A Retrospective Study

**DOI:** 10.1155/2017/4935397

**Published:** 2017-08-27

**Authors:** Fatima Vally, Jeffrey Presneill, Thomas Cade

**Affiliations:** ^1^Department of Maternal Fetal Medicine, Pregnancy Research Centre, Royal Women's Hospital, 20 Flemington Road, Parkville, Melbourne, VIC, Australia; ^2^The Royal Melbourne Hospital, City Campus, 300 Grattan St, Melbourne, VIC, Australia; ^3^Department of Obstetrics and Gynaecology, The University of Melbourne, Melbourne, VIC, Australia

## Abstract

**Background:**

Current data on the rates of macrosomia in women with gestational diabetes mellitus (GDM) are heterogenous. No study has specifically examined macrosomia rates in women with diet-controlled gestational diabetes.

**Aims:**

To compare the rates of macrosomia between mothers with diet-controlled GDM to mothers without diabetes mellitus.

**Methods:**

A retrospective study in which all patients with diet-controlled GDM and singleton pregnancies in 2014 were considered for inclusion in the study. These cases were individually matched to mothers without GDM and without type 1 or 2 diabetes. Cases were matched to parity, age, and BMI. Controls were selected from the same year and as close as possible to the date of delivery of the case. Primary outcomes were macrosomia, defined by estimated fetal weight >90th centile and >95th centile (separately).

**Results:**

The estimated adjusted odds ratio for the presence of maternal GDM in the presence of EFW > 90th percentile (adjusted for maternal age, BMI, gravidity, parity, baby gender, and EGA) was 0.63 (95% CI 0.30–1.3; *P* = 0.21). The estimated adjusted odds ratio for the association of maternal GDM and EFW > 95th percentile was 0.66 (95% CI 0.26–1.7; *P* = 0.38).

**Conclusions:**

Our findings suggest that macrosomia is not increased in women with diet-controlled GDM. The study registration number is AQA 16/01.

## 1. Introduction

Current data on the rates of macrosomia in gestational diabetes are heterogeneous, highlighted particularly by the inconsistent definitions of true macrosomia. There is also a lack of data specifically examining macrosomia rates in gestational diabetes patients whose diabetes is controlled by diet alone.

Common definitions of macrosomia use threshold birthweight percentiles (e.g., 90th, 95th, and 97th percentiles) or birthweights (e.g., 4000 g, 4500 g) associated with an increase in neonatal adverse outcomes thought to be due to birthweight [1]. Women with gestational diabetes may undergo extra surveillance in the third trimester because of traditional concern around macrosomia. However, the true risk of large babies in treated patients with milder degrees of hyperglycaemia is not well described.

The present study aimed to evaluate the proportion of macrosomia defined according to Australian growth chart values in women with gestational diabetes mellitus (GDM) treated only with dietary measures, using a retrospective analysis, in order to identify whether the babies of these women had a higher risk of macrosomia compared to a matched “low-risk” obstetric population without diabetes mellitus.

## 2. Materials and Methods

We compared a cohort of patients with gestational diabetes managed by dietary measures with a concurrent pregnant control group matched for age, parity, and body mass index (BMI). All patients with diet-controlled gestational diabetes with singleton pregnancies in a single calendar year at the antenatal clinics of the Royal Women's Hospital, Melbourne, were considered for inclusion in the study. Patients were identified using data generated from the hospital's electronic medical record system. Patients with multiple pregnancies were excluded, as were patients whose body mass index was unrecorded.

Gestational diabetes was diagnosed using the 1998 Australasian Diabetes in Pregnancy Society (ADIPS) criteria, whereby patients underwent a 75 g nonfasting glucose challenge test [2]. Patients with a 1-hour blood glucose ≥ 8.0 mmol/L underwent a fasting 75 g glucose tolerance test. Gestational diabetes mellitus was diagnosed in patients with a fasting glucose ≥ 5.5 mmol/L or a 2-hour glucose ≥ 8.0 mmol/L [3]. All participants in this study undertook the glucose tolerance test in the third trimester.

Identified patients with gestational diabetes attended a group class with a dietician, a diabetes educator, and a physiotherapist to learn appropriate dietary carbohydrate intake, lifestyle modification, and blood glucose level monitoring via a Freestyle Lite (Abbott Diabetes Care Inc.) or Performa (Roche) glucometer. Patients were instructed to measure their blood glucose every morning after an overnight fast and two hours after the commencement of breakfast, lunch, and dinner. These levels were recorded in a handbook. Satisfactory control was defined as both a fasting glucose of less than 5.0 mmol/L and a postprandial glucose of less than 6.7 mmol/L. Insulin therapy was commenced if three elevated readings at the same time of day were recorded within one week despite adequate dietary modifications. All patients had subsequent phone consultations with a diabetes educator and a follow-up individual consultation with a dietician. Diet-controlled GDM patients were seen fortnightly from the time of diagnosis of GDM until 36-week gestation, then weekly from 36-week gestation until delivery.

Control patients were selected from those without gestational type 1 or type 2 diabetes attending for obstetric care at the same hospital. Cases and controls were matched for parity (exactly if possible, otherwise nearest available), age (within mutually exclusive five-year groups partitioning the clinic patient age range, 20–24, 25–29, 30–34, 35–39, 40–44, and 45–49), and a similar partitioning of the clinic BMI range (within 5 units, 16–20, 21–25, 26–30, 31–35, 36–40, 41–45, 46–50, and 51–55). Controls were selected from the same calendar year (2014), with a date of delivery as close as possible to the date of delivery of the gestational diabetic case patient.

The study primary outcome was the differential proportion of patients with macrosomia between cases and controls. Two separate thresholds for macrosomia were studied, defined by estimated fetal weights above the 90th centile and 95th centiles, using Australian Birth Weight charts generated from a cross-sectional population-based study of 2.53 million singleton live births in Australia between 1998 and 2007 [4].

### 2.1. Statistical Analyses

Summary statistics were reported as mean with standard deviation and/or median, interquartile range (IQR) and range, as appropriate. The closeness of 1 : 1 matching between cases and controls was assessed as differences within matched pairs of maternal age, BMI, and parity. Matched case control gestational diabetes data were analysed with regression models using conditional logistic regression models which returned estimated adjusted odds ratios for the presence of (variously defined) macrosomia associated with maternal gestational diabetes, adjusted for maternal age, BMI, gravidity, parity, baby gender, and estimated gestational age (EGA) [5]. Data were analysed using Stata version 14.2 [6]. No adjustments were made for multiple comparisons.

The adequacy of specification of fitted adjusted conditional logistic models was evaluated with link tests and case-wise diagnostic measures of leverage, lack of fit, and influence [5].

The study was approved as a registered audit by the Royal Women's Hospital Human Research Ethics Committee and the need for individual patient consent was waived. Authors had full access to all of the data in the study. Funding was not required for the study.

## 3. Results

### 3.1. Maternal Characteristics

217 cases were identified out of a total of 7185 births in a single calendar year. There were 15 exclusions, seven with twin pregnancies and eight without a BMI recorded, leaving 202 cases as singleton pregnancies with diet-controlled gestational diabetes. These cases were matched 1 : 1 using parity, age, BMI, and date of delivery, to an equal number of 202 control nondiabetic obstetric patients from the same women's hospital in the same year.

Across age, BMI, and parity, the number of cases and controls in each stratum were very similar (Tables 1, 2, and 3).

### 3.2. Neonatal Characteristics

Of the neonates delivered by the 202 maternal gestational diabetic cases, 110 were male and 92 female. In the control group, 100 of the 202 neonates were male and 102 were female.

Within this group of 404 babies the median estimated gestational age (EGA) was 39 weeks (IQR 38 to 40, range 25 to 42 weeks; mean 38, SD 2.75 weeks); and the median baby weight was 3288 g (IQR 2914 to 3620, range 525 to 4895 g; mean 3191, SD 708 g). There was a mean EGA difference of 0.8 weeks (95% CI 0.4 to 1.2 weeks; *P* < 0.001 paired *t* test) between GDM and non-GDM groups, with the mean EGA in children of mothers without diabetes exceeding that of mothers with gestational diabetes.

Likewise, there was a mean difference of 221 g (95% CI 106 g to 336 g; *P* < 0.001 paired *t* test), with the mean birthweight in children of mothers without diabetes exceeding that of mothers with gestational diabetes. For every extra week of EGA, the adjusted odds ratio for gestational diabetes was 0.8 (95% CI 0.71–0.91; *P* < 0.001), meaning that, adjusting for the influence of other variables in the model, there was strong evidence that gestational diabetes was less likely (odds ratio less than one) with higher EGA. Thus there is strong evidence of relatively small mean differences in birth weight and EGA between babies of mothers with and without gestational diabetes.

### 3.3. Prevalence of Macrosomia

The number of neonates in the case and control groups meeting definitions of macrosomia based on birth weights above the 90th and 95th percentiles of normal estimated fetal weights (EFW) is shown in Table 4.

The estimated adjusted odds ratio for the presence of maternal gestational diabetes in the presence of EFW > 90th percentile (adjusted for maternal age, BMI, gravidity, parity, baby gender, and EGA) was 0.63 (95% CI 0.30–1.3; *P* = 0.21).

In a similar logistic model using the same variables, the estimated adjusted odds ratio for the association of maternal gestational diabetes and macrosomia defined above the 95th percentile was 0.66 (95% CI 0.26–1.7; *P* = 0.38). These estimates did not show important change when two high influence and two high leverage matched pairs were excluded.

## 4. Discussion

Risk factors previously reported for macrosomia include elevated maternal BMI, diabetes mellitus, multiparity, and gestational age > 40 weeks [7]. Macrosomia may complicate up to 20–30% of patients with GDM based on heterogenous data, but most macrosomic babies are born to women without GDM [8]. In the HAPO study, the frequency of macrosomia was found to be increased in GDM by 50% compared to non-GDM mothers. The macrosomia rate in nonobese, nondiabetic mothers was 6.7% and, in nonobese women with GDM, the rate was 10.2% [3]. Studies have examined the rates of macrosomia in GDM, but no study has specifically looked at the rates of macrosomia in diet-controlled GDM.

The present retrospective study has shown that rates of macrosomia are not increased in women with diet-controlled GDM, compared to women without GDM, and showed adjusted odds ratio for maternal BMI, parity, age, and estimated gestational age. We aimed to evaluate the strength of the previously known association of maternal diabetes with macrosomia. The data from the present matched study, which sampled patients at a large Australian obstetric hospital clinic in a single calendar year, do not support a strong association between diet-controlled GDM as defined using the 1998 ADIPS criteria and macrosomic babies, whether defined as either above the 90th or 95th percentiles of Australian national birthweights.

The Australian national birthweight percentiles were generated from data on 2.53 million singleton live births between 1998 and 2007 [4]. These remain the most specific Australian data on the observed range of birthweight.

Although macrosomia confers well-known risks to both the mother and neonate, assessment of this association is complicated by variation in the definitions of macrosomia [9]. Common definitions are birthweights above the 90th, 95th, or 97th percentiles for gestational age, or alternatively simple binary birthweight thresholds, such as above 4000 g or 4500 g [10]. A retrospective analysis of 34685 large-for-gestational-age and adequate-for-gestational-age babies born at term between 2004 and 2008 found an association between adverse outcomes and birth weights > 4000 g [9]. However, neonatal adverse outcomes were also seen for babies born <4000 g who were large-for-gestational-age, suggesting that birthweight centiles may be more useful in defining macrosomia. A randomized controlled trial comparing induction of labour with expectant management for large-for-date fetuses for prevention of neonatal morbidity suggested a biologically relevant definition of macrosomia to be fetal weights > 95th percentile, with increased neonatal adverse outcomes observed at that threshold [11].

The present study, conducted in 2014, employed the Australasian Diabetes in Pregnancy Society (ADIPS) criteria, originally described in 1998, to diagnose GDM [3]. In 2015, the diagnostic criteria for GDM changed to those of the International Association of Diabetes and Pregnancy Study Groups (IADPSG), though these new criteria have not been universally adopted. These latest criteria define GDM at one or more of the following elevated glucose levels around a 75 g fasting glucose tolerance test: fasting ≥ 5.1 mmol/L, 1 hour ≥ 10.0 mmol/L and 2 hour ≥ 8.5 mmol/L [12].

These new IADPSG criteria were based on a subanalysis of the HAPO trial, which examined the relationship between cases of hyperglycaemia milder than diabetes mellitus and macrosomia (birth weight > 90th percentile), neonatal hypoglycaemia, primary caesarean delivery, and fetal hyperinsulinism (cord C peptide > 90th percentile) [12, 13]. These new criteria largely relate to fetal size in a population of untreated mothers. It is estimated that the IADPSG criteria may increase the number of women diagnosed with GDM by 35% relative to previous criteria with these newly diagnosed GDM cases being less marked cases of hyperglycaemia [14]. These milder cases of hyperglycaemia may be more likely to be controlled by dietary measures. Further prospective studies are required to examine whether the adoption of the new IADPSG criteria will be accompanied by increased rates of macrosomia. Further prospective studies are required to examine whether these women require extra ultrasound surveillance for macrosomia and whether they may be triaged into lower risk care pathways.

*Limitations*. Limitations of the study include that it is a retrospective study and a substantially larger data set would be required to be more certain as to the relationship between diet-controlled GDM and macrosomia and to undertake further regression analyses exploring potential predictors of macrosomia. A larger data set would also be required to explore other less frequent outcomes related to macrosomia, including shoulder dystocia and Erb's palsy.

Other factors that influence macrosomia include ethnicity of the parents, smoking, weight gain during pregnancy, and previous macrosomic babies. These are potential confounding factors, particularly as significant weight gain during pregnancy and a prior history of macrosomia predispose to macrosomia. Smoking has a well-known association with growth-restriction and may have masked the potential for macrosomia in mothers who smoke. It is known that the normal distribution of birthweight varies across ethnicities; therefore what is considered macrosomic in one ethnicity may be considered a normal birthweight in another. This study did not take these factors into account and we acknowledge this as a limitation of the study.

## 5. Conclusions

In conclusion, the present study suggests that macrosomia is not more common in women with diet-controlled GDM. Consideration should be given to conducting a prospective study with larger numbers to draw robust conclusions about the rates of macrosomia in diet-controlled GDM. Consideration should also be given as to whether extra ultrasound surveillance is required in these cases, especially if these women attend appropriate initial consultations with a diabetes educator and dietician, and particularly if they are linked in with an experienced maternity centre and if care-givers are familiar with blood glucose targets.

## Figures and Tables

**Table 1:** Distribution of age across cases and controls.

Age range (y)	Cases (*n* = 202)	Controls (*n* = 202)
15–19	0	0
20–24	12	12
25–29	59	58
30–34	78	79
35–39	35	36
40–44	17	16
45–49	1	1

Median age of all 404 obstetric patients: 31 y, interquartile range (IQR): 28 to 35 y, range 20 to 46 y; mean 31.7 y, standard deviation (SD) 5.04. Of the 202 pairs of maternal ages, the median difference between cases and controls was 0 years (IQR −2 to 1 years, range −4 to 4 years).

**Table 2:** Distribution of body mass index across cases and controls.

BMI (kg/m^2^)	Cases (*n* = 202)	Controls (*n* = 202)
16–20	28	26
21–25	93	96
26–30	47	47
31–35	17	16
36–40	10	10
41–45	5	5
46–50	1	1
51–55	1	1

Median BMI of all 404 obstetric patients: 24, IQR 22 to 28, range 17 to 52; mean BMI 25.8, SD 5.92. Of the 202 pairs of maternal body mass index (BMI) values, the median difference between cases and controls was 0, IQR −1 to 1, range −6 to 11.

**Table 3:** Distribution of parity across cases and controls.

Parity	Cases (*n* = 202)	Controls (*n* = 202)
0	115	115
1	54	53
2	21	22
3	9	9
4	1	1
5	1	0
6	1	1
7	0	0
8	0	1

Median parity of all 404 obstetric patients in this study was 0, IQR 0 to 1, range 0 to 8.

**Table 4:** Number and proportion of macrosomic neonates at two diagnosis thresholds in case and control groups.

	*N* = 202	*N* = 202	Case-Control
Estimated fetal weight	Cases, *n* (%)	Controls, *n* (%)	Percent (95% CI)
>90%	16 (7.9)	21 (10.4)	−2.5 (−8.9 to 3.9)^†^
>95%	10 (5.0)	11 (5.4)	−0.5 (−5.4 to 4.4)^‡^

^†^Unadjusted odds ratio 0.76 (95% exact CI 0.37 to 1.53), exact McNemar *P* = 0.51; ^‡^unadjusted odds ratio 0.91 (95% exact CI 0.35 to 2.4), exact McNemar *P* = 0.83.
